# Assessing the role of FET-PET imaging in glioblastoma recurrence: A retrospective analysis of diagnostic accuracy

**DOI:** 10.1016/j.bas.2025.105599

**Published:** 2025-09-08

**Authors:** Lisa S. Hönikl, Claire Delbridge, Igor Yakushev, Chiara Negwer, Denise Bernhardt, Friederike Schmidt-Graf, Bernhard Meyer, Arthur Wagner

**Affiliations:** a Department of Neurosurgery, Technical University Munich, School of Medicine and Health, Germany; b Department of Neuropathology, Technical University Munich, School of Medicine and Health, Germany; c Department of Nuclear Medicine, Technical University Munich, School of Medicine and Health, Germany; d Department of Radiotherapy, Technical University Munich, School of Medicine and Health, Germany; e Department of Neurology, Technical University Munich, School of Medicine and Health, Germany

**Keywords:** FET-PET imaging, GBM recurrence, Specificity and sensitivity

## Abstract

**Introduction:**

Glioblastoma (GBM) is the most aggressive primary brain tumor in adults, with a high recurrence rate. Differentiating tumor progression from treatment-induced changes remains a diagnostic challenge.

**Research question:**

Can FET-PET reliably distinguish true GBM recurrence from post-therapeutic effects across multiple recurrence stages, and how does MGMT promoter methylation influence diagnostic accuracy?

**Material and methods:**

We performed a retrospective, single-center study correlating PET findings with histopathological results from surgery or biopsy at first, second, and third recurrence. Sensitivity and specificity were calculated using three interpretive scenarios for uncertain PET results. Subgroup analysis based on MGMT methylation was also performed.

**Results:**

960 patients with GBM were treated at our department between 2006 and 2021, of whom 347 (36.1 %) had one tumor recurrence during follow-up with 156 (45.0 %) FET-PET scans available. 95 patients (9.9 %) had a second recurrence with a FET-PET conducted in 37 of these (39.0 %), whereas 23 patients (2.4 %) had a third recurrence with a FET-PET available in 8 patients (34.8 %). Sensitivity was 95 % at first recurrence, 96 % at second, and 83 % at third. Specificity was low overall (13 %, 0 %, 0 %). No statistically robust differences in diagnostic performance were observed between MGMT subgroups (p > 0.4 for all).

**Discussion and conclusion:**

PET imaging demonstrated high sensitivity for detecting GBM recurrence but showed variable specificity. Notably, sensitivity declined with an increasing number of recurrences, suggesting that cumulative treatment effects and greater tumor heterogeneity over time may affect diagnostic performance. Further prospective studies are needed to refine diagnostic thresholds and improve clinical decision-making.

## Highlights

• FET-PET shows high sensitivity in detecting glioblastoma recurrence.
• Sensitivity decreases with increasing number of tumor recurrences.
• Specificity remains low across all recurrence stages.
• Diagnostic performance is independent of MGMT promoter methylation.

## 1 Introduction

Glioblastoma (GBM), classified as CNS WHO grade 4 (Louis et al., 2021), is the most aggressive and common primary brain tumor in adults. Despite advances in multimodal treatment strategies, including surgical resection followed by radiation and chemotherapy according to the Stupp protocol, the prognosis for patients remains poor, with a median survival of approximately 14–16 months (Stupp et al., 2005, 2017; Vogel et al., 2023). Tumor recurrence is almost inevitable and presents significant challenges for both diagnosis and treatment (Vaz-Salgado, 2023). One of the critical hurdles in managing recurrent GBM is distinguishing true tumor recurrence from treatment-induced changes, such as radiation necrosis or pseudoprogression (Jacob et al., 2023; Kruser et al., 2013). Accurate diagnosis is essential for guiding subsequent therapeutic decisions, as unnecessary treatments or interventions can significantly impact patient outcomes and quality of life.

18-fluoride-fluoro-ethyl-tyrosine positron emission tomography (FET-PET) imaging has emerged as a promising tool to boost diagnostic accuracy by providing metabolic insights that complement conventional MRI, particularly when MRI findings are inconclusive (Lindsey et al., 2020; Wang et al., 2025; Ameya D Puranik et al., 2019; Arpita Sahu et al., 2023). FET-PET provides metabolic and molecular information that complements conventional anatomical imaging modalities such as MRI. This is particularly valuable in cases where MRI findings are inconclusive due to overlapping features of recurrence and treatment-related effects (Bobek-Billewicz et al., 2023; Jajodia et al., 2022). While prior studies have suggested the utility of FET-PET in identifying recurrent GBM, its diagnostic performance, specifically in terms of sensitivity and specificity, remains under debate. Furthermore, the role of PET imaging in routine clinical practice has yet to be fully established, particularly in consideration of its significant economic burden on health care providers.

This retrospective, single-center study aims to evaluate the role of FET-PET imaging in detecting glioblastoma recurrence by analyzing its diagnostic accuracy. Ultimately, this study seeks to contribute to the growing body of evidence supporting the diagnostic accuracy of FET-PET imaging in glioblastoma recurrence.

## 2 Methods

### 2.1 Study cohort

All patients included in the study had available formalin-fixed, paraffin-embedded (FFPE) tumor specimens and underwent surgical resection followed by adjuvant radiation and chemotherapy according to the Stupp protocol (Stupp et al., 2005). Patients formed a consecutive series, as all eligible glioblastoma cases with histopathological confirmation and available FET-PET imaging during the defined study period were included. Inclusion criteria mandated that the tumor resection was performed at our institution, that a minimum follow-up period of three months was available, and that the diagnosis of GBM was confirmed by histopathological examination. Patients were excluded if a definitive tumor recurrence was not confirmed by histopathology, if they had any other grade 4 tumor harboring an isocitrate dehydrogenase (IDH) mutation, or if they did not undergo PET imaging at the time of suspected tumor recurrence.

### 2.2 Study design

This is a retrospective, single-center study aimed to evaluate the diagnostic accuracy of FET-PET imaging in detecting GBM recurrence. For the primary outcome, FET-PET imaging findings obtained during clinical suspicion of recurrence were compared with histopathological results from re-resection or biopsy, enabling the calculation of sensitivity and specificity. For a stratified analysis, three groups were examined to calculate sensitivity and specificity: in the first group (1), only cases with clearly defined FET-PET results—either positive or negative—aligned with histopathologically confirmed recurrence or its exclusion were included, with uncertain cases excluded. In the second group (2), uncertain cases were classified as negative, whereas in the third group (3), uncertain cases were assigned to the positive group. This approach allowed for a comprehensive evaluation of the impact of uncertain PET findings on diagnostic accuracy.

Secondary outcome parameters included stratification by MGMT status, time to recurrence, survival data, as well as consecutive rates of surgery or other forms of treatment.

### 2.3 PET imaging and evaluation

Patients received an intravenous injection of 185–222 MBq of 18F-fluoroethyltyrosine (FET). FET-PET imaging was performed as a static acquisition, with image acquisition taking place between 20 and 40 min after intravenous tracer injection. FET-PET scans were evaluated as part of clinical routine by board-certified nuclear medicine physicians. Interpretation combined visual assessment and semi-quantitative evaluation (tumour-to-background ratio, TBR), but no prospectively fixed numeric cut-off was applied. Final reports categorized findings into “tumor”, “no tumor”, or “uncertain”.

The median interval between FET-PET and histopathological confirmation was 9 days (range 0–115). Histopathological analysis of resection or biopsy specimens served as the reference standard. Tissue samples were obtained during subsequent surgery or biopsy following PET imaging. All specimens were assessed by board-certified neuropathologists according to WHO CNS classification criteria. Pathologists were blinded to the PET findings and categorized cases as tumor recurrence (positive) or treatment-related changes (negative).

### 2.4 Statistics

Data analysis was performed using Microsoft Excel and R (Version 1.4.1717). Sensitivity and specificity were calculated from 2 × 2 contingency tables to evaluate the diagnostic accuracy of PET imaging in detecting glioblastoma recurrence. In addition, positive predictive value (PPV), negative predictive value (NPV), and likelihood ratios (LR+ and LR−) were determined to provide a more comprehensive measure of diagnostic performance. For all accuracy estimates, 95 % confidence intervals were calculated using the Clopper–Pearson method for proportions. This approach allowed us to account for variability and to demonstrate the robustness of the outcome measures across recurrence stages. Because only categorical outcomes were available, ROC curves and AUC could not be generated retrospectively. Inter-reader agreement was not formally assessed.

MGMT promoter methylation status was available only for a subset of patients and strata were small and imbalanced across recurrence stages. Therefore, MGMT-stratified analyses were pre-specified as exploratory and limited to descriptive comparisons (proportions with 95 % CIs). Fisher's exact tests were reported where numerically feasible, but no multivariable modelling was performed due to sample size constraints and missingness.

### 2.5 Ethical considerations

This study was conducted in compliance with the ethical standards of the Declaration of Helsinki. Approval was obtained from the local ethics committee prior to the initiation of the study (approval number 740/20). Given the retrospective nature of the research, the ethics committee granted a waiver for patient informed consent.

## 3 Results

### 3.1 Patient cohort

**Fig. 1:**
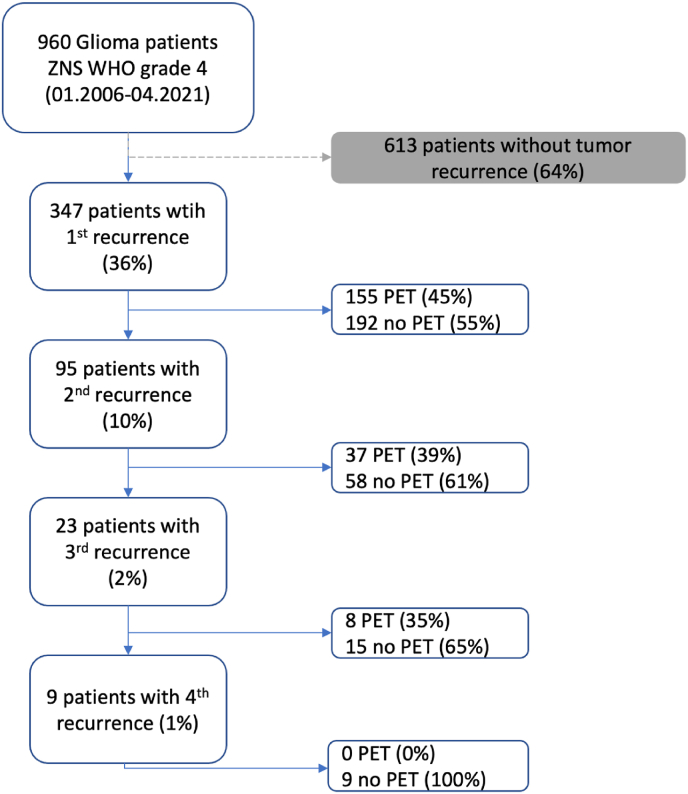
Flow chart of patient cohort recruitment. PET – Positron Emission Tomography.

Between 2006 and 2021, a total of 960 glioblastoma (GBM) patients were screened. Of these, 613 patients were excluded due to a lack of documented tumor recurrence. This resulted in a study cohort of 347 patients (36 %) with a first recurrence. Among them, 155 patients (45 %) underwent PET imaging, while 192 (55 %) did not. A second recurrence was documented in 95 patients (10 %), with 37 patients (39 %) receiving PET imaging and 58 (61 %) without PET. A third recurrence was recorded in 23 patients (2 %), of whom 8 (35 %) underwent PET imaging, whereas 15 (65 %) did not. For patients experiencing a fourth recurrence (9 patients; 1 %), none underwent PET imaging (Fig. 1).

The study cohort consisted of 347 patients, including 124 women (36 %) and 223 men (64 %, Table 1). The age at diagnosis ranged from 10 to 90 years, with a median age of 57 years. Overall survival ranging from 11 to 4712 days, with a median survival of 555 days. Women had a median overall survival of 647 days, while men had a median survival of 528 days. The number of recurrence surgeries ranged from 1 to 5, with a median of 1 surgery for both genders.

**Table 1:** Patient characteristics (age, sex, overall survival) across the study cohort and FET-PET subgroups at first, second, and third recurrence.

	Age (years)	Sex	Overall survival (days)
Study cohort n347	57 (10–90)	64 % male 36 % female	555 (11–4712)
1. recurrence n155	58 (10–84)	61 % male 39 % female	589 (20–4712)
2. recurrence n37	51 (14–78)	65 % male 35 % female	1370 (371–4712)
3. recurrence n8	50 (42–65)	63 % male 37 % female	1241 (382–2310)

Among the 155 patients with FET-PET at first recurrence, the median age was 58 years (10–84), with a sex distribution of 61 % male and 39 % female, and a median overall survival of 589 days (20–4712). In the subgroup with second recurrence (n = 37), patients had a median age of 51 years (14–78), 65 % were male, and median survival reached 1370 days (371–4712). For patients with FET-PET at third recurrence (n = 8), the median age was 50 years (42–65), 63 % were male, and the median survival was 1241 days (382–2310).

No adverse events related to PET imaging or the histopathological reference standard were reported in the available records.

### 3.2 Representative cases: correlation of MRI, FET-PET, and histopathology

**Fig. 2:**
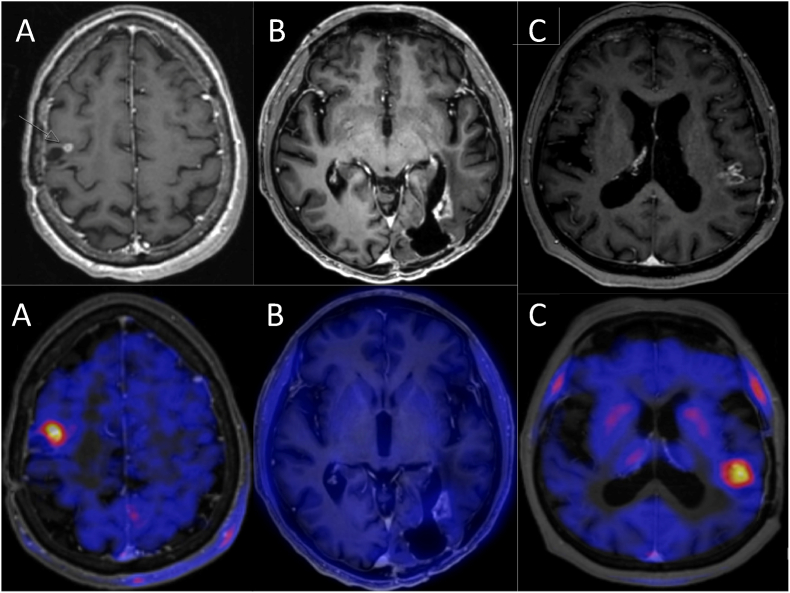
Representative MRI (A1–C1) and corresponding FET-PET images (A2–C2) of three patients with suspected recurrence. Case A: PET positive, histopathology confirmed tumor recurrence. Case B: PET negative, no tumor detected histologically. Case C: PET positive.

To illustrate the diagnostic role of FET-PET in clinical decision-making, Fig. 2 presents three representative cases with corresponding MRI (A1–C1) and FET-PET images (A2–C2). In all cases, MRI initially suggested tumor recurrence, prompting further evaluation with FET-PET imaging. In case A, FET-PET showed a clear tracer uptake (A2), which was confirmed as true tumor recurrence by histopathology. In case B, no relevant FET uptake was observed (B2), and histopathological analysis likewise revealed no active tumor tissue, indicating a treatment-related change. In contrast, case C showed positive tracer uptake on FET-PET (C2), but histopathological workup did not confirm tumor recurrence. These examples highlight the added value—as well as the limitations—of FET-PET in differentiating true recurrence from therapy-induced changes.

### 3.3 First recurrence

The first suspected recurrence requiring subsequent surgical intervention occurred at a median of 277 days (range: 10–3338 days) after initial diagnosis. The first FET-PET scan indicating tumor recurrence was performed at a median of 244 days (range: 0–2381 days) following completion of radiotherapy. The evaluation of FET-PET diagnostic accuracy in the first recurrence was conducted based on the three predefined groups (Table 2).

**Table 2:** Diagnostic accuracy of FET-PET in detecting glioblastoma recurrence across the first, second, and third recurrence, stratified by three classification scenarios (1 = uncertain cases excluded, 2 = uncertain cases counted as negative, 3 = uncertain cases counted as positive). Histopathology (HP) served as the reference standard. Sensitivity (Sens), specificity (Spec), positive predictive value (PPV), negative predictive value (NPV), and likelihood ratios (LR+ and LR–) are reported. Values marked as NE (not estimable) indicate that calculations were not possible due to absent cases in a category (e.g., no true negatives). Values marked as inf (infinite) indicate that the calculation yielded an infinite result, typically when no false positives occurred in the dataset.

Recurrence	Scenario	a (TP)	b (FP)	c (FN)	d (TN)	Sensitivity	Sens 95 % CI	Specificity	Spec 95 % CI	PPV	PPV 95 % CI	NPV	NPV 95 % CI	LR+	LR-
1st recurrence	Scenario 1	122	7	6	1	95 %	[90.1 %, 98.3 %]	12.5 %	[0.3 %, 52.7 %]	94.6 %	[89.1 %, 97.8 %]	14.3 %	[0.4 %, 57.9 %]	1.09	0.38
1st recurrence	Scenario 2	122	7	17	9	89 %	[81.1 %, 92.7 %]	56 %	[29.9 %, 80.2 %]	94.6 %	[89.1 %, 97.8 %]	34.6 %	[17.2 %, 55.7 %]	2.01	0.22
1st recurrence	Scenario 3	135	11	8	1	94 %	[89.3 %, 97.6 %]	8 %	[0.2 %, 38.5 %]	92.5 %	[86.9 %, 96.2 %]	11.1 %	[0.3 %, 48.2 %]	1.03	0.67
2nd recurrence	Scenario 1	25	2	1	0	96 %	[80.4 %, 99.9 %]	0 %	[0.0 %, 84.2 %]	92.6 %	[75.7 %, 99.1 %]	0.0 %	[0.0 %, 97.5 %]	0.96	Inf
2nd recurrence	Scenario 2	25	3	5	4	83 %	[65.3 %, 94.4 %]	57 %	[18.4 %, 90.1 %]	89.3 %	[71.8 %, 97.7 %]	44.4 %	[13.7 %, 78.8 %]	1.94	0.29
2nd recurrence	Scenario 3	31	5	6	0	84 %	[68.0 %, 93.8 %]	0 %	[0.0 %, 52.2 %]	86.1 %	[70.5 %, 95.3 %]	0.0 %	[0.0 %, 45.9 %]	0.84	Inf
3rd recurrence	Scenario 1	5	0	1	0	83 %	[35.9 %, 99.6 %]	NE	NE	100.0 %	[47.8 %, 100.0 %]	0.0 %	[0.0 %, 97.5 %]	NE	NE
3rd recurrence	Scenario 2	5	1	2	0	71 %	[29.0 %, 96.3 %]	0 %	[0.0 %, 97.5 %]	83.3 %	[35.9 %, 99.6 %]	0.0 %	[0.0 %, 84.2 %]	0.71	Inf
3rd recurrence	Scenario 3	7	0	1	0	88 %	[47.3 %, 99.7 %]	NE	NE	100.0 %	[59.0 %, 100.0 %]	0.0 %	[0.0 %, 97.5 %]	NE	NE

In Group 1, only confirmed FET-PET positive or negative cases with corresponding histopathological (HP) confirmation were included. The analysis yielded a sensitivity of 95 % (95 % CI: 90.1 %–98.3 %) and a specificity of 12.5 % (95 % CI: 0.3 %–52.7 %) based on the following distribution: 122 HP-positive cases were correctly identified as FET-PET positive (a), while 6 HP-positive cases were FET-PET negative (c). Among the HP-negative cases, 7 were misclassified as FET-PET positive (b), and only 1 was correctly classified as FET-PET negative (d).

In Group 2, uncertain cases were assigned to the negative group, leading to a sensitivity of 89 % (95 % CI: 81.1 %–92.7 %) and a specificity of 56 % (95 % CI: 29.9 %–80.2 %). Here, 122 HP-positive cases were correctly classified as FET-PET positive (a), while 17 HP-positive cases were classified as negative (c). Among the HP-negative cases, 7 were misclassified as FET-PET positive (b), whereas 9 were correctly identified as negative (d).

In Group 3, uncertain cases were counted as positive, resulting in a sensitivity of 94 % (95 % CI: 89.3 %–97.6 %) and a specificity of 8 % (95 % CI: 0.2 %–38.5 %). In this scenario, 135 HP-positive cases were identified as FET-PET positive (a), while 8 HP-positive cases were FET-PET negative (c). Among the HP-negative cases, 11 were misclassified as positive (b), and only 1 was correctly classified as negative (d).

### 3.4 Second recurrence

The evaluation of FET-PET imaging in the detection of a second glioblastoma recurrence followed the same classification into three groups (Table 2). The second suspected recurrence occurred at a median of 975 days (range: 256–3816 days) after initial diagnosis. The corresponding FET-PET imaging in case of suspected tumor recurrence was performed at a median of 340 days (range: 35–3756 days) following the last radiotherapy. For the second glioblastoma recurrence, FET-PET imaging demonstrated a high sensitivity but variable specificity depending on case classification.

In Group 1, sensitivity was 96 % (95 % CI: 80.4 %–99.9 %), while specificity remained 0 % (95 % CI: 0.0 %–84.2 %). Among 26 HP-positive cases, 25 were correctly classified as FET-PET positive, while 2 HP-negative cases were misclassified as positive.

In Group 2, where uncertain cases were counted as negative, sensitivity decreased to 83 % (95 % CI: 65.3 %–94.4 %), while specificity improved to 57 % (95 % CI: 18.4 %–90.1 %).

In Group 3, with uncertain cases classified as positive, sensitivity was 84 % (95 % CI: 68.0 %–93.8 %), but specificity remained at 0 % (95 % CI: 0.0 %–52.2 %).

These findings indicate that FET-PET imaging retained a high sensitivity in detecting a second glioblastoma recurrence but showed substantial limitations in specificity depending on how uncertain cases were classified. Notably, specificity remained 0 % in Group 1 and Group 3, highlighting the challenge of distinguishing true recurrence from treatment-related changes at this stage of disease progression.

### 3.5 Third recurrence

For the third glioblastoma recurrence, FET-PET sensitivity remained high, but specificity was consistently 0 % across all groups (Table 2). The third suspected recurrence occurred at a median of 910.5 days (range: 264–1973 days) after initial diagnosis. The corresponding FET-PET imaging was performed at a median of 159.5 days (range: 32–1005 days) following the last radiotherapy.

In Group 1, sensitivity was 83 % (95 % CI: 35.9 %–99.6 %), with 5 correctly classified HP-positive cases and 1 misclassified as negative. No HP-negative cases were correctly identified.

In Group 2, where uncertain cases were classified as negative, sensitivity dropped to 71 % (95 % CI: 29.0 %–96.3 %), but specificity remained 0 % (95 % CI: 0.0 %–97.5 %).

In Group 3, with uncertain cases counted as positive, sensitivity increased to 88 % (95 % CI: 47.3 %–99.7 %), though specificity remained 0 %.

These findings reinforce that while FET-PET maintains good sensitivity for detecting a third recurrence, its specificity is extremely limited at this stage of disease progression.

### 3.6 Molecular correlation of specificity and sensitivity with MGMT status

Exploratory stratification by MGMT promoter methylation status did not demonstrate consistent or statistically robust differences in the diagnostic performance of FET-PET. Sensitivity estimates appeared similar between methylated and unmethylated tumors, although confidence intervals were wide due to small and imbalanced groups (methylated: Sensitivity 92.9 % (95 % CI 66.1–99.8), unmethylated: Sensitivity 96.3 % (95 % CI 89.7–99.2), p = 0.498 for the first recurrence; methylated: Sensitivity 91.7 % (95 % CI 61.5–99.8), unmethylated: Sensitivity 100 % (95 % CI 59.0–100), p = 1.0 for the second recurrence). Specificity estimates also showed no detectable differences between molecular subtypes (p = 1.0 for both the first (methylated: Specificity 33.3 % (95 % CI 0.8–90.6), unmethylated: Specificity 25.0 % (95 % CI 0.6–80.6)) and second recurrence (methylated: Specificity 0.0 % (95 % CI 0.0–97.5), unmethylated: Specificity 0.0 % (95 % CI 0.0–97.5)). Given the limited sample size and missing MGMT data, these results should be interpreted with caution and considered descriptive rather than definitive.

## 4 Discussion

The findings of this study demonstrate that FET-PET imaging exhibits high sensitivity for differentiating glioblastoma recurrence from pseudoprogression or therapy-induced changes, particularly during the early stages of recurrence. However, our data indicate a significant decline in sensitivity with successive recurrences, suggesting that the diagnostic challenge of differentiating true tumor regrowth from treatment-related alterations becomes increasingly difficult over time. To our knowledge, few studies have systematically analyzed the diagnostic performance of FET-PET across multiple recurrences with direct histopathological correlation focusing exclusively on glioblastoma patients. These results underscore the utility of FET-PET in the initial recurrence setting while highlighting the need for further research to optimize diagnostic strategies in later recurrences. In cases of PET negativity, especially when only small or equivocal lesions are present on MRI, abstaining from immediate surgery and opting for close radiological follow-up may be a reasonable approach. This strategy can help avoid unnecessary procedures in patients without viable tumor tissue. Nevertheless, operative decisions should not rely solely on PET findings but must be made in the context of the overall clinical picture and multidisciplinary assessment.

For the first recurrence, FET-PET demonstrated robust diagnostic performance, with sensitivities ranging from 89 % to 95 % across the different classification groups, suggesting that in patients with suspected first recurrence, FET-PET provides a valid means of identifying viable tumor tissue and guiding further therapeutic decisions. However, for the second recurrence, sensitivity declined, particularly when uncertain cases were categorized as negative (83 % sensitivity in Group 2). This trend continued in the third recurrence, where the lowest sensitivity was observed in Group 2 (71 %). These findings suggest that cumulative treatment effects, such as radiation necrosis, gliosis, and therapy-induced metabolic changes, increasingly obscure the ability of FET-PET to distinguish between tumor progression and non-tumorous changes. As a result, the risk of false-negative findings rises with each recurrence, necessitating a careful interpretation of imaging results in later-stage disease.

While sensitivity remained consistently high across all groups in our cohort, specificity was highly dependent on how uncertain cases were classified, showing the greatest increase when these cases were counted as negative. Our definition of PET positivity reflects real-world reporting practice, relying on combined visual and semi-quantitative interpretation without a fixed numeric threshold. While this enhances external validity, it precluded the reconstruction of ROC curves and AUC. To improve transparency, we provided complete 2 × 2 contingency tables, predictive values, likelihood ratios, and confidence intervals, which allow robust interpretation of diagnostic performance. The three analytic scenarios were intended as sensitivity analyses to illustrate the impact of different adjudications of “uncertain” cases, rather than post hoc optimization. Nevertheless, the lack of prespecified thresholds and inter-reader agreement should be considered and prospective studies will be required to more precisely define and standardize the evaluation of PET findings.

A further limitation relates to the reference standard. Although histopathological confirmation represents the gold standard, the neuropathologists were aware that tissue sampling was performed under the suspicion of tumor recurrence. While they did not have access to PET results, complete blinding to the clinical context was not possible and may have influenced diagnostic classification.

These observations align with previous research on the diagnostic performance of FET-PET in high-grade gliomas (Puranik et al., 2024). A large retrospective study by Singnurkar et al. found an overall sensitivity of 91 % and specificity of 84 % for static FET-PET parameters in detecting tumor recurrence (Singnurkar et al., 2023). Similarly, Cui et al. reported pooled values of 88 % sensitivity and 78 % specificity across 15 studies (Cui et al., 2021). The most recent study also demonstrated an overall sensitivity and specificity with 91.6 and 76.9 % respectively, with a diagnostic accuracy of 87.13 % (Puranik et al., 2024). These findings are consistent with our results in early recurrences, where FET-PET showed high sensitivity but limited specificity. However, our study highlights an important limitation of FET-PET in later-stage recurrences, where diagnostic accuracy declines, likely due to an increased presence of non-tumorous changes mimicking active tumor tissue.

The results from de Zwart et al. (de Zwart, 2020), who analyzed 10 studies on FET-PET with a pooled sensitivity of 90 % and specificity of 86 %, further reinforce the clinical value of this imaging modality. In contrast, our study found that specificity remained consistently low across all recurrence stages, emphasizing the limited ability of FET-PET to rule out recurrence, particularly in cases with treatment-related alterations.

While previous studies have suggested that MGMT methylation status may influence recurrence patterns as detected by FET-PET, our findings did not observe consistent or statistically robust differences in sensitivity or specificity across MGMT data (Niyazi et al., 2012). MGMT promoter methylation status had no significant impact on the sensitivity or specificity of FET-PET. This aligns with findings from previous studies indicating that amino acid PET tracers, including FET, function independently of molecular tumor markers such as MGMT. Unlike contrast agents for MRIs, which rely on blood-brain barrier disruption and may be influenced by tumor microenvironment changes, FET uptake is mediated through the LAT1 transporter and reflects active glioma metabolism, regardless of MGMT status (Miyagawa et al., 1998; Habermeier et al., 2015). This independence makes FET-PET an attractive imaging modality for both methylated and unmethylated glioblastomas, ensuring broad applicability across patient subgroups.

## 5 Limitations

As is the nature of retrospective data analysis, our study cannot confer causality from the results presented herein, and the completeness and accuracy of clinical information is subject to documentation available within hospital data systems. A central limitation is verification bias: only surgically treated patients with histological confirmation could be included in the diagnostic accuracy analysis. Consequently, cases with negative PET findings, or those judged as negative on MRI without subsequent surgery, could not be integrated. This methodological constraint inevitably inflates sensitivity estimates and reduces specificity, as truly negative cases are underrepresented and cannot be formally accounted for in a retrospective design.

Furthermore, repeated recurrences within the same patients were treated as independent observations. While this introduces a certain dependency within the data, it also represents a strength of the study, as it reflects the longitudinal follow-up of individual patients across multiple recurrence stages. This allows assessment of how diagnostic accuracy evolves over time and provides insights into preoperative decision-making in increasingly complex clinical situations.

A key limitation is that PET positivity was defined according to routine clinical reports, which combined visual and semi-quantitative interpretation but did not apply a pre-specified numeric cut-off. As a result, ROC analyses and inter-reader reliability could not be assessed. Subgroup analyses stratified by MGMT promoter methylation status were constrained by missing data and small, imbalanced group sizes, particularly in later recurrences, and should therefore be interpreted with caution. Finally, the present work focused on diagnostic accuracy rather than therapeutic impact; thus, no direct conclusions can be drawn about the influence of FET-PET on treatment decisions or patient outcomes.

Our study is further limited by the fact that neuropathologists were not blinded in a strict sense. Although they did not have access to PET imaging, they were informed that tissue was obtained under the assumption of tumor progression, which may have introduced bias in histopathological classification.

## 6 Conclusion

FET-PET imaging is a highly sensitive method for detecting glioblastoma recurrence, particularly in early stages. However, sensitivity declines as the number of recurrences increases, likely due to the accumulation of treatment-related changes that complicate differentiation from active tumor tissue. No consistent differences by MGMT status were observed in this exploratory, underpowered subgroup analysis. Given the observed limitations, a multimodal diagnostic approach incorporating additional imaging and molecular techniques may be necessary in later-stage recurrences to optimize clinical decision-making.

## Statement of ethics and consent to participate

The presented study meets the ethical standards outlined in the Declaration of Helsinki, ethics approval was obtained, and the favorable vote was registered under the number 740/20. In this retrospective study, only patients who were already deceased at the time of the study were included. Informed consent for study participation was therefore no longer possible.

## Availability of data and materials

The datasets used and/or analyzed during the current study are available from the corresponding author on reasonable request.

## Funding

This research did not receive any specific funding or financial support from public, commercial, or not-for-profit organizations.

## Conflict of interest

All authors report no conflict of interest concerning the materials or methods used in this study or the findings specified in this publication.
